# Globally Relaxed Selection and Local Adaptation in *Boechera stricta*

**DOI:** 10.1093/gbe/evac043

**Published:** 2022-03-29

**Authors:** Yi-Ye Liang, Xue-Yan Chen, Biao-Feng Zhou, Thomas Mitchell-Olds, Baosheng Wang

**Affiliations:** 1 Key Laboratory of Plant Resources Conservation and Sustainable Utilization, South China Botanical Garden, Chinese Academy of Sciences, Guangzhou, China; 2 University of the Chinese Academy of Sciences, Beijing, China; 3 Department of Biology, Duke University, Durham, North Carolina, USA; 4 Center of Conservation Biology, Core Botanical Gardens, Chinese Academy of Sciences, Guangzhou, China

**Keywords:** adaptive evolution, distribution of fitness effects, effective population size, GC-biased gene conversion, conserved non-coding region, *Boechera*

## Abstract

The strength of selection varies among populations and across the genome, but the determinants of efficacy of selection remain unclear. In this study, we used whole-genome sequencing data from 467 *Boechera stricta* accessions to quantify the strength of selection and characterize the pattern of local adaptation. We found low genetic diversity on 0-fold degenerate sites and conserved non-coding sites, indicating functional constraints on these regions. The estimated distribution of fitness effects and the proportion of fixed substitutions suggest relaxed negative and positive selection in *B. stricta*. Among the four population groups, the NOR and WES groups have smaller effective population size (*N_e_*), higher proportions of effectively neutral sites, and lower rates of adaptive evolution compared with UTA and COL groups, reflecting the effect of *N_e_* on the efficacy of natural selection. We also found weaker selection on GC-biased sites compared with GC-conservative (unbiased) sites, suggested that GC-biased gene conversion has affected the strength of selection in *B. stricta*. We found mixed evidence for the role of the recombination rate on the efficacy of selection. The positive and negative selection was stronger in high-recombination regions compared with low-recombination regions in COL but not in other groups. By scanning the genome, we found different subsets of selected genes suggesting differential adaptation among *B. stricta* groups. These results show that differences in effective population size, nucleotide composition, and recombination rate are important determinants of the efficacy of selection. This study enriches our understanding of the roles of natural selection and local adaptation in shaping genomic variation.

**Significance**

Natural selection shapes the landscape of genomic variation, but we know little about how evolutionary forces affect the strength of selection across populations and genomic regions. In this study, we quantify the strength of selection in 467 *Boechera stricta* accessions based on whole-genome sequencing data and reveal relaxed selection in this species due to long-term small effective population size. We also found varied strengths of selection among genomic regions, influenced by nucleotide composition, recombination rate, and the genetic basis of adaptation.

## Introduction

Understanding the relative importance of selective and stochastic processes influencing genetic variation across the genome is a fundamental goal in evolutionary biology (Mitchell-Olds et al. 2007; Vitti et al. 2013). Recent studies suggest that both positive selection favoring beneficial alleles and negative selection against deleterious alleles shape the landscape of genomic diversity (Hough et al. 2013; Williamson et al. 2014; Kern and Hahn 2018; O'Connor et al. 2019). For example, positive selection has fixed substantial fractions of substitutions in many plants, animals, and insects (Bierne and Eyre-Walker 2004; Halligan et al. 2010; Slotte et al. 2010; Williamson et al. 2014; Lin et al. 2018). Negative selection has purged large-effect mutations controlling complex traits (O'Connor et al. 2019) and kept new mutations at low frequency in human populations (Prohaska et al. 2019; Schoech et al. 2019). Evidence that rare variants have a great impact on gene expression suggests that pervasive purifying selection has acted on *cis*-acting regulatory variants in humans (Hernandez et al. 2019). The strength of positive and negative selection varies among species (Gossmann et al. 2010; Grivet et al. 2017; Lin et al. 2018; Zhao et al. 2020), and also varies across different portions of the genome (Williamson et al. 2014; Josephs et al. 2017; Uricchio et al. 2019; Horvath et al. 2021).

Many evolutionary forces can affect the strength of selection. Effective population size (*N_e_*) is one of the primary determinants of the efficacy of selection, because the strength of selection on a mutation (*N_e_s*) is a function of *N_e_* and the selection coefficient (*s*) (Kimura 1983). Therefore, natural selection is expected to be prevalent in species with large *N_e_*, and relaxed in species with small *N_e_*. Consistent with this expectation, high levels of adaptive divergence have been detected in species with large *N_e_*, for example, *Drosophila* (Bierne and Eyre-Walker 2004), rodents (Halligan et al. 2010), poplar (Lin et al. 2018), oaks (Liang et al. 2021), and the outcrossing crucifer *Capsella grandiflora* (Slotte et al. 2010; Williamson et al. 2014; Josephs et al. 2017; Horvath et al. 2021), whereas low levels of adaptive divergence are found in hominids (Eyre-Walker and Keightley 2009) and *Arabidopsis thaliana* (Foxe et al. 2008; Hämälä and Tiffin 2020), which have small *N_e_*. A positive correlation between the rates of adaptive evolution and *N_e_* was also observed in plants and animals, suggesting significant effects of *N_e_* in determining the level of adaptive divergence (Strasburg et al. 2011; Galtier 2016; Nam et al. 2017). Studies on the distribution of fitness effects (DFE) of new mutations have revealed that species with small *N_e_* have relatively high proportions of mutations that are effectively neutral (Gossmann et al. 2010; Arunkumar et al. 2015; Laenen et al. 2018), indicating a clear influence of *N_e_* on the strength of negative selection.

In addition to variation among species, the strength of selection varies among genomic regions. It is hypothesized that low-recombination regions experienced stronger interference between selected loci and lower efficacy of selection, relative to high-recombination regions (Hill and Robertson 1966; Felsenstein 1974). This positive correlation between the efficacy of selection and recombination rate has been found in some species, for example, birds (Corcoran et al. 2017), *Drosophila* (Campos et al. 2014), and domesticated rice (Flowers et al. 2011); but not in others, for example, *Arabidopsis* (Slotte et al. 2011) and wild rice (Flowers et al. 2011). It is also expected that selection mainly acts in coding regions and the evolution of intergenic regions is usually neutral (Hough et al. 2013). However, studies have detected strong signals of selection in conserved non-coding sequences (CNSs) (Andolfatto 2005; Kousathanas et al. 2011; Haudry et al. 2013; Williamson et al. 2014). To date, the CNSs were only characterized in a few species, for example, *Drosophila* (Andolfatto 2005), human (Davydov et al. 2010; Lindblad-Toh et al. 2011), rodents (Kousathanas et al. 2011), and Brassicaceae species (Haudry et al. 2013; Williamson et al. 2014; Horvath et al. 2021). We know little about the strength and extent of selection on CNS, as well as the relative importance of selection acting in CNS versus coding regions.

GC-biased gene conversion (gBGC) also can influence the rate of protein evolution (Bolivar et al. 2018; Hämälä and Tiffin 2020). During meiotic recombination, gBGC promotes conversion to G or C with strong (“S”) hydrogen bonds over A or T with weak (“W”) hydrogen bonds, regardless of their fitness effects (Mugal et al. 2015). This process results in apparent relaxed selection on substitutions from W to S (“WS”) in *A. thaliana* and related species (Hämälä and Tiffin 2020) and Flycatchers (Bolivar et al. 2018). Strong selection may act on locally beneficial alleles and drive adaptation to the local environment, that is, local adaptation. For example, a mutation on flowering time gene *SVP* has contributed to the adaptation of *A. thaliana* in the Yangtze River basin, but not in other geographic regions (Zou et al. 2017). For species distributed in heterogeneous habitats, many of these locally adaptive mutations would be polymorphic at the species-level, generating a signature of globally weak positive and negative selection. Because many of these evolutionary forces can influence signals of adaptive evolution in the genome (Gossmann et al. 2010; Hämälä and Tiffin 2020), accurately quantifying the strength of selection and dissecting determinants of the efficacy of selection remain challenging.

*Boechera stricta* (Brassicaceae), a pre-dominantly inbreeding species (inbreeding coefficient *F_IS_* = 0.9) (Song et al. 2006), is distributed across much of western North America (Mitchell-Olds 2001; Song et al. 2006; Rushworth et al. 2011). Populations of *B. stricta* occupy diverse habitats (Lee and Mitchell-Olds 2011; Rushworth et al. 2011). Previous studies in *B. stricta* revealed two subspecies (Song et al. 2009), which were further divided into four genetically and geographically differentiated groups (Wang et al. 2019a). Groups COL and UTA are found primarily in the states of Colorado and Utah. The WES and NOR groups occur in both Montana and Idaho, including a partial zone of sympatry (supplementary fig. S1, Supplementary Material online) (Wang et al. 2019a). Substructure was found in COL and UTA groups, but genetic differentiation between subgroups was relatively lower than among these four major groups (Wang et al. 2019a). Population, quantitative, and ecological genetics approaches have investigated the genetic basis of adaptive evolution in natural habitats of *B. stricta*. These studies detected a set of quantitative trait loci (QTLs) for ecologically important traits (Anderson et al. 2011, 2014; Rushworth et al. 2011; Prasad et al. 2012; Lee and Mitchell-Olds 2013; Lee et al. 2017; Arisz et al. 2018; Carley et al. 2021; Lin et al. 2021; Yan et al. 2021), and suggested that both antagonistic pleiotropy and conditional neutrality may have contributed to local adaptation (Anderson et al. 2013). A recent study sequenced whole genomes of ∼500 genotypes of *B. stricta*, and demonstrated that the pattern of genomic variation was affected by long-term balancing selection and sorting of ancestral balanced polymorphisms (Wang et al. 2019a). Extensive genomic resources in *B. stricta* and its close relationship to *A. thaliana* offer opportunities to investigate the role of natural selection driving divergence and speciation. However, our current knowledge of selection in *B. stricta* is largely based on particular traits or genes, and we know little about genome-wide selection and complex interactions between selection and other evolutionary forces in shaping genetic variation.

In this study, we applied multiple population genomic and statistical approaches to investigate the determinants of the efficacy of selection in *B. stricta*. First, we quantified the strength of positive and negative selection in both coding and CNS regions of the four *B. stricta* population groups. To do this, we sampled 467 genotypes representing the four genetic groups, inferred the DFE of mutations based on genome-wide single nucleotide polymorphism (SNP) data, and calculated the proportion of adaptive substitutions. Next, we investigated how effective population size, gBGC and recombination rate have influenced the efficacy of selection in *B. stricta*. Finally, we scanned the genome for signatures of selective sweeps, and investigated the genetic architecture of local adaptation.

## Results

### Pattern of Genetic Variation Across the Genome of *B. stricta*

**Fig. 1.**
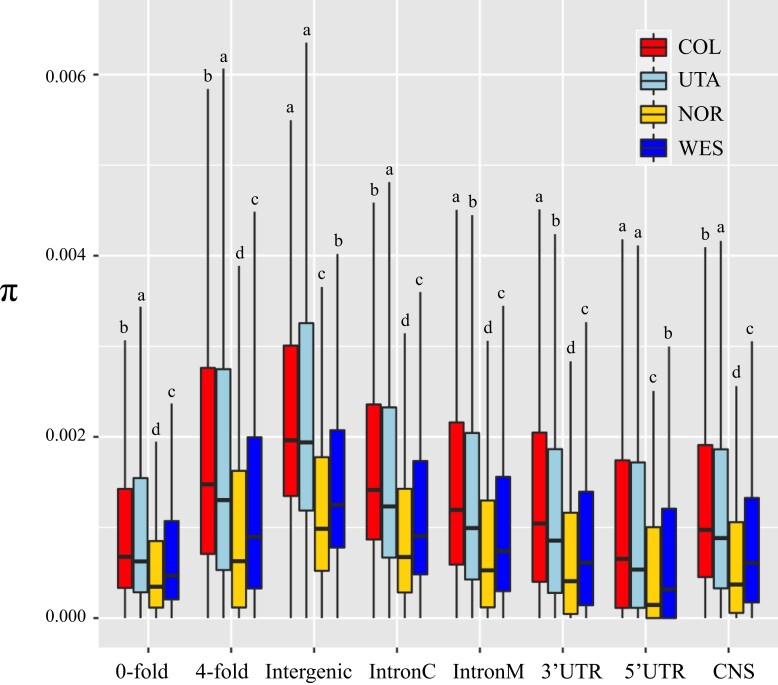
Nucleotide diversity (*π*) at each category of sites in four genetic groups of *Boechera stricta*. In these box plots, the median is shown by a horizontal line, while the bottom and top of each box represents the first and third quartiles. The whiskers extend to 1.5 times the interquartile range. Outliers are not shown in the plot. Different letters (a–d) above each box indicate significant differences among groups based on Mann–Whitney *U* tests with Bonferroni correction (*P* < 0.05). IntronC, middle region after excluding the first and last 30 bp of each intron; IntronM, the first and last 30 bp of each intron; CNS, conserved non-coding sequence.

Previous study detected four genetic groups (WES, COL, UTA, and NOR) in *B. stricta* (Wang et al. 2019a) (supplementary fig. S1, Supplementary Material online). Here, we focused on 467 individuals representing the four groups (supplementary table S1, Supplementary Material online). All re-sequencing data used in this study have been previously published (Wang et al. 2019a, 2019b). After filtering low-quality data, we retained 3,995,289 SNPs with high confidence for subsequent analyses (see Materials and Methods). To investigate the pattern of genomic diversity in *B. stricta*, we classified genomic sites as 0-fold degenerate, 4-fold degenerate, intronic, 5′ UTR, 3′ UTR, or intergenic based on annotation of the *B. stricta* reference genome (Lee et al. 2017). In all groups, high levels of genetic diversity (*π*) were detected in 4-fold degenerate sites (range from 1.54 × 10^−3^ to 2.69 × 10^−3^) and intergenic regions (*π* = 1.48 × 10^−3^–2.70 × 10^−3^), while lower levels of diversity were found in 0-fold degenerate sites (range from 0.94 × 10^−3^ to 1.68 × 10^−3^) and 5′ UTRs (range from 0.83 × 10^−3^ to 1.36 × 10^−3^; *W* ranges from 7,982,764 to 55,805,673, *P* < 0.05, Mann–Whitney *U* tests with Bonferroni correction; fig. 1 and supplementary table S2, Supplementary Material online), reflecting evolutionary constraint in functional regions. To investigate whether non-coding regions are also under selection, we conducted GERP++ analysis (Davydov et al. 2010) to identify conserved sequences (CSs) in the *B. stricta* genome based on a 10-way multiple alignment of Brassicaceae species’ assemblies (supplementary table S3, Supplementary Material online). We found 208,881 CSs with length >12 bp, totaling 21.3 Mb (11.8% of the assembled *B. stricta* genome), in which 100,585 CSs, totaling 6.2 Mb (29% of total length of CSs) were located outside of protein-coding regions (CNS). Similar to what is observed in *A. thaliana* (Haudry et al. 2013), we found a higher proportion of conserved sites in 5′ UTR and 3′ UTR (13.7% and 11.2%, respectively) as well as in intronic regions flanking exons (7.8% within 30 bp of slice sites), compared with the center of introns (3.4%; supplementary fig. S2, Supplementary Material online). The proportion of conserved sites was generally low in intergenic regions, although a large proportion of intergenic sites (12.7–15.9%) located within 100 bp of genic regions were conserved (supplementary fig. S2, Supplementary Material online). Estimated genetic diversity (*π* = 0.93 × 10^−3^–1.65 × 10^−3^) in CNSs was lower than estimates in most categories of sites (*W* ranges from 12,710,361 to 30,343,007, *P* < 0.05, Mann–Whitney *U* tests with Bonferroni correction; fig. 1 and supplementary table S2, Supplementary Material online), indicating functional constraints in CNSs. Among groups, WES and NOR groups showed lower diversity than COL and UTA in most site categories (*W* ranges from 6,527,471 to 57,836,388, *P* < 0.05, Mann–Whitney *U* tests with Bonferroni correction; fig. 1 and supplementary table S2, Supplementary Material online).

### Strength of Purifying and Positive Selection in *B. stricta*

**Fig. 2.**
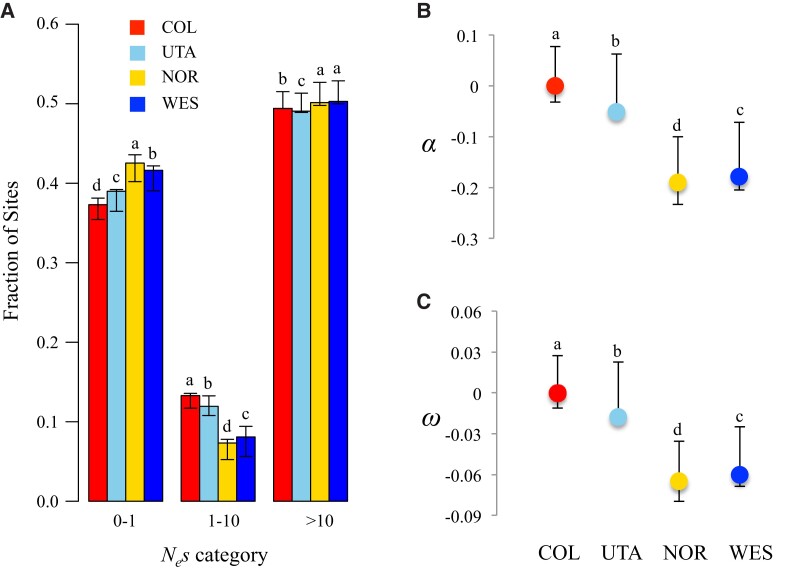
Negative and positive selection in the *Boechera stricta* genome. (*A*) Comparisons of the DFE among *B. stricta* groups at 0-fold sites. (*B*) The proportion of adaptive substitutions (*α*), and (*C*) the rate of adaptive substitution (*ω*) of 0-fold sites in each group. Different letters (a–d) above each bar indicate significant differences among groups based on Mann–Whitney *U* tests with Bonferroni correction (*P* < 0.05).

The heterogeneous patterns of genomic variation in *B. stricta* may be due to varied strength of selection across the genome. To test this hypothesis, we quantified the amount of negative and positive selection acting on different categories of sites. We calculated DFE of new mutations to assess the efficacy of negative selection. To do that, we used the method of Keightley and Eyre-Walker (2007) to compare the site frequency spectrum (SFS) and divergence of various site categories versus SFS for 4-fold degenerate sites from non-conserved genomic regions, which are putatively neutral. Estimates of DFE showed a remarkably consistent picture among the four population groups. For all groups, negative selection was much stronger in coding regions than non-coding regions (supplementary table S4*A*, Supplementary Material online), in accordance with the patterns of diversity described above. The estimated DFE revealed that 37−42% of the polymorphic 0-fold sites were effectively neutral (*N_e_s* < 1), while 60–100% of non-coding mutations were neutral (fig. 2*A*, supplementary table S4*A*, Supplementary Material online). Among the four population groups, we found a higher frequency of weakly deleterious mutations that behave as effectively neutral in NOR (42.5%) and WES (41.6%) groups, compared with COL (37.3%) and UTA (39.0%) groups (*W* ranges from 6 to 265, *P* < 2e^−16^, Mann–Whitney *U* tests with Bonferroni correction; fig. 2*A*, supplementary table S4*B*, Supplementary Material online). When we looked at 0-fold sites from conserved regions, we found smaller proportions of effectively neutral sites (21.8−26.5%) and larger proportions of strongly deleterious mutations (65.9−67.1%; supplementary table S4*A*, Supplementary Material online), compared with 0-fold sites as whole, suggesting stronger purifying selection in conserved coding regions. The proportion of strongly deleterious mutations in CNS regions was also higher than non-coding regions as a whole, but lower than in 0-fold sites (*W* ranges from 25,938 to 38,945, *P* < 5e^−6^, Mann–Whitney *U* tests with Bonferroni correction; supplementary table S4*A*, Supplementary Material online).

To further quantify the strength of positive selection in *B. stricta*, we applied three complementary methods. First, we used DFE-alpha (Keightley and Eyre-Walker 2007; Eyre-Walker and Keightley 2009) to estimate the proportion of fixations driven by positive selection (*α*) and the relative rate of adaptive substitution (*ω*). To control the effects of slightly deleterious mutations to estimate *α*, DFE-alpha predicts the number of substitutions contributed by deleterious mutations based on inferred DFE, and then compares the predicted substitutions with observed data (Eyre-Walker and Keightley 2009). The *α* was defined as *α* = 1−(*p_N_*/*p_S_*)/(*d_N_*/*d_S_*), where p*_N_*/p*_S_* is the ratio of non-synonymous compared with synonymous polymorphism, and *d_N_*/*d_S_* is the ratio of non-synonymous to synonymous divergence (Smith and Eyre-Walker 2002). The *ω* is the rate of adaptive substitution relative to synonymous substitutions, *ω* = *α* × (*d_N_*/*d_S_*) (Eyre-Walker and Keightley 2009). Positive values of *α* and *ω* represent strong positive selection driving substitutions, while negative or near-zero values (due to excess of non-synonymous polymorphisms) represent weak selection (Eyre-Walker and Keightley 2009; Hahn 2018). Second, we applied asymptoticMK (Haller and Messer 2017) to calculate asymptotic estimates of *α*, while considering the effect of deleterious mutations and demographic history. We also estimated *α* using a modification of the McDonald–Kreitman (MK) test (Fay 2011). To minimize the bias due to slightly deleterious mutations, we excluded polymorphisms with derived allele frequency <15%. Because all three methods generated similar estimates of *α*, we only present results of DFE-alpha in the main text, and provide all results in supplementary table S4*A* and *B*, Supplementary Material online. We found limited evidence of adaptive evolution in *B. stricta*. The estimated *α* and *ω* values at 0-fold sites were negative in all groups (fig. 2*B* and *C*; supplementary table S4*A* and *B*, Supplementary Material online). When considering only 0-fold sites from conserved genomic regions, we obtained positive estimates of *α* and *ω* in groups COL (*α* = 0.198, *ω* = 0.049) and UTA (*α* = 0.228, *ω* = 0.058), but not in WES (*α* = −0.022, *ω* = 0.056) or NOR (*α* = −0.014, *ω* = −0.034; supplementary table S4*A* and *B*, Supplementary Material online).

### Effects of gBGC and Recombination Rate on the Efficacy of Selection

**Fig. 3.**
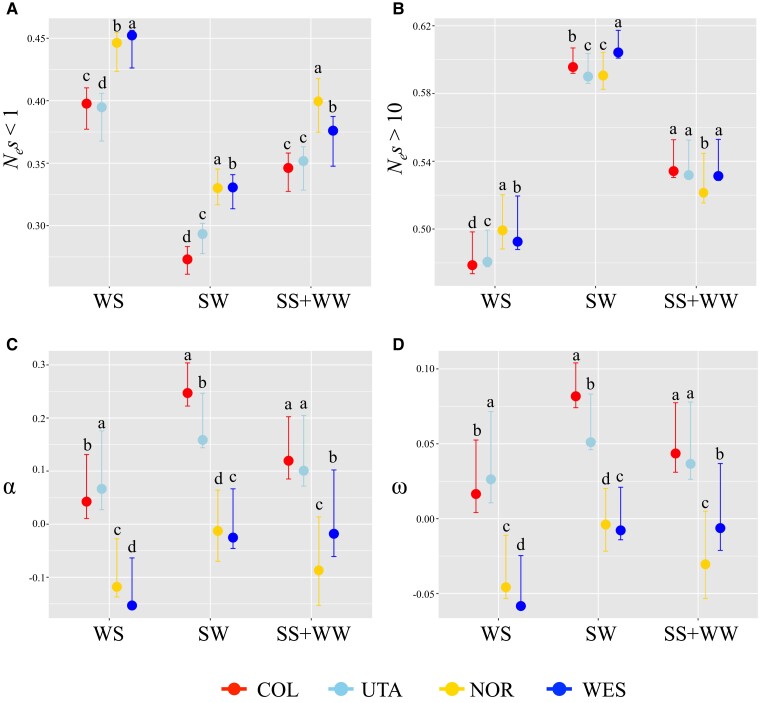
Effects of gBGC on the efficacy of selection in four *Boechera stricta* groups. Four statistics and 95% confidence intervals (indicated by error bar) were estimated for different mutation categories: WS (ancestral allele A or T, derived allele G or C; A/T → G/C), SW (ancestral allele G or C, derived allele A or T; G/C → A/T), and WW + SS (ancestral and derived allele are both G or C or both A or T; A/T → A/T; and GC → GC). (*A*) The proportion of effectively neutral sites (*N_e_s* < 1). (*B*) The proportion of strongly deleterious mutations (*N_e_s* > 10). (*C*) The proportion of adaptive substitution (*α*). (*D*) The rate of adaptive substitution (*ω*). Different letters (a–d) above each bar indicate significant differences among groups based on Mann–Whitney *U* tests with Bonferroni correction (*P* < 0.05).

gBGC is expected to influence the efficacy of selection. To investigate how gBGC constrains selection in *B. stricta*, we estimated DFE for three mutation categories SW, WS, and GC-conservative (SS + WW) sites (see Materials and Methods). The WS sites have more, and SW sites have fewer, non-synonymous sites in the nearly neutral category (*N_e_s* < 1) than GC-conservative sites (SS + WW) in all four groups (*W* ranges from 18 to 40,000, *P* < 2e^−16^, Mann–Whitney *U* tests with Bonferroni correction; fig. 3*A*, supplementary table S4*A*, Supplementary Material online), suggesting relaxed selection in WS sites due to gBGC. For GC-conservative (“unbiased”) sites, we found a higher frequency of weakly deleterious mutations that behave as effectively neutral (*N_e_s* < 1) in NOR (39.9%) and WES (37.6%) population groups, compared with COL (34.6%) and UTA (35.1%; *W* ranges from 1,625 to 2,260, *P* < 2e^−16^, Mann–Whitney *U* tests with Bonferroni correction; fig. 3*A*, table S4*B*, Supplementary Material online). The gBGC also influenced the estimates of *α* and *ω* in *B. stricta.* For GC-conserved sites, we found positive *α* and *ω* in COL (*α* = 0.119, *ω* = 0.043) and UTA (*α* = 0.101, *ω* = 0.037), and the WS sites have lower, and SW sites have higher *α* and *ω* than GC-conservative sites (*W* ranges from 230 to 37,544, *P* < 5e^−5^, Mann–Whitney *U* tests with Bonferroni correction; fig. 3*C* and *D*, supplementary table S4*A*, Supplementary Material online). The estimated *α* and *ω* were negative for all site categories in WES and NOR groups (fig. 3*C* and *D*; supplementary table S4*A* and *B*, Supplementary Material online).

The efficacy of selection may be correlated with recombination rate across the genome. Hill–Robertson interference (HRI) (Hill and Robertson 1966; Felsenstein 1974) predicts weak selection on regions of low recombination rate due to prevalent interference between selected loci. To analyze the influence of recombination rate on strength of selection in *B. stricta*, we divided GC-conservative sites into three equal-size bins based on recombination rate (see Materials and Methods). To account for possible effects of gBGC on the efficacy of selection, we considered only GC-conservative sites in this analysis. We found mixed evidence for the HRI theory. In the COL group, we found a lower proportion of weakly deleterious mutations (*N_e_s* < 1), higher *α* and *ω* in bins of high recombination rate, compared with bins of low recombination (*W* ranges from 9,781 to 36,517, *P* < 2e^−16^, Mann–Whitney *U* tests with Bonferroni correction; supplementary table S5, Supplementary Material online), consistent with the expectation of the HRI theory. However, in the other three groups, higher proportions of weakly deleterious mutations (*N_e_s* < 1) were detected in high-recombination regions than in regions of low-recombination rate (*W* ranges from 13,390 to 34,440, *P* < 2e^−16^, Mann–Whitney *U* tests with Bonferroni correction; supplementary table S5, Supplementary Material online). In the UTA group, efficacy of positive selection was slightly higher in regions of high recombination rate than in regions with low recombination rates (*α* = 0.076 vs. 0.066 and 0.053; *ω* = 0.027 vs. 0.025 and 0.018; *W* = 1,162, *P* < 2e^−16^ and *W* = 2,631, *P* < 2e^−16^ for *α* and *ω*, respectively; Mann–Whitney *U* tests with Bonferroni correction; supplementary table S5, Supplementary Material online). Negative values of *α* and *ω* were detected in all bins with different recombination rate in groups NOR and WES (supplementary table S5, Supplementary Material online).

### Genomic Signature of Local Adaptation in *B. stricta*

**Fig. 4.**
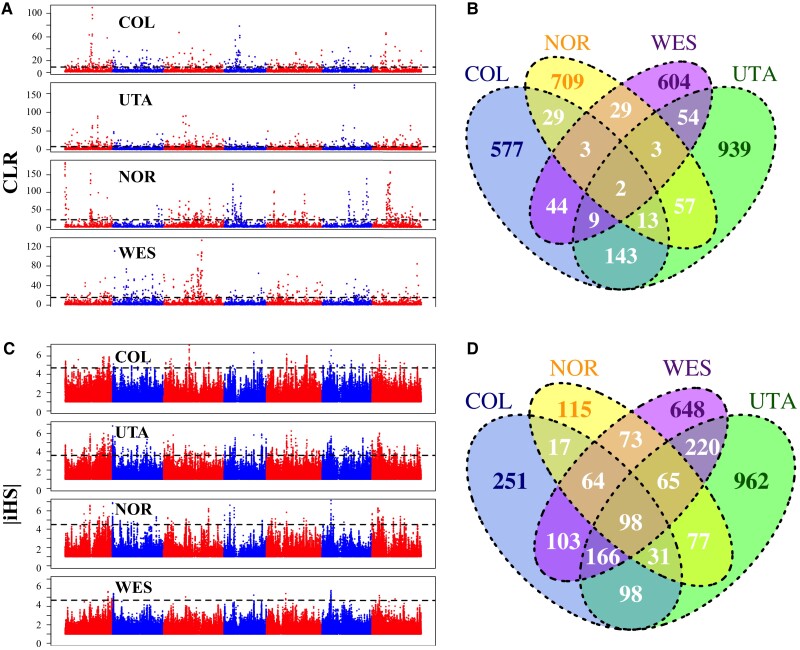
Signatures of positive selection in *Boechera stricta*. (*A*) Manhattan plot of CLR values estimated in each group. (*B*) Number of group-specific and group-shared genes located in swept regions. (*C*) Manhattan plot of absolute values of iHS. (*D*) Number of group-specific and group-shared candidate genes identified by iHS. In *A* and *C*, alternative colors paint the different chromosomes, and dashed horizontal lines mark the significance threshold (FDR < 0.01).

Although the efficacy of selection is relative low across the genome of *B. stricta*, natural selection may have acted at a small set of genes and promoted populations to adapt to local environments, leaving genetic signatures of local adaptation. In each group we used two approaches to scan the genome for evidence of local adaptation. We first performed a composite likelihood ratio (CLR) test using *SweepFinder2* (DeGiorgio et al. 2016) for identifying recent selective sweeps. *SweepFinder2* identified 3.5−5.1% of the *B. stricta* genome as outliers (swept regions; fig. 4*A*). Compared with genomic background, these swept regions show multiple signatures of selection, including reduced nucleotide diversity (*π*), elevated frequency of derived alleles (more negative Fay & Wu’s *H*), and an excess of low frequency alleles (more negative Tajima’s *D*); (*W* ranges from 188,314 to 810,149, *P* = 0.03 − 4.60 × 10^−92^, Mann–Whitney *U* test; see details in supplementary table S6, Supplementary Material online). In comparisons between a focal region (showing a sweep signal) versus other regions, swept regions also show greater differentiation (*F_ST_*) than the rest of the genome (with few exceptions; supplementary table S6, Supplementary Material online). There are 820, 1,220, 845, and 748 genes embedded in these swept regions, identified as candidates under selection in groups COL, UTA, NOR, and WES, respectively. Most of these candidate swept genes were lineage specific, with 67.9−83.9% of candidates only detected in one group (fig. 4*B*). The gene ontology (GO) analyses revealed that candidate genes in the WES group were enriched in an ADP binding function (false discovery rate, FDR = 0.0086; supplementary table S7, Supplementary Material online). Candidates in other groups were not enriched in any GO categories (FDR > 0.05; supplementary table S7, Supplementary Material online), suggesting a diverse set of functional genes may have influenced adaptive evolution in *B. stricta*.

We also calculated integrated haplotype score (iHS) to track decay of haplotype homozygosity for haplotypes extending from each SNP with MAF > 0.05. We found 1,078–2,533 outlier SNPs (0.18−0.50% of all tested SNPs) showing significantly higher or lower iHS (fig. 4*C*), compared with neutral model expectations (FDR < 0.01), and considered 828, 1,717, 540, and 1,437 genes within 10-kb of the outlier SNPs as candidates under selection in COL, UTA, NOR, and WES groups, respectively. Similar to the findings in *SweepFinder*2, most candidate genes identified by iHS were specific to a single-population group (fig. 4*D*). Notably, genomic regions surrounding these iHS outlier SNPs showed higher diversity, Tajima’s *D*, and Fay & Wu’s *H*, and lower *F_ST_* than genomic background (*W* ranges from 549,869 to 2,322,823, *P* = 0.039−5.775 × 10^−26^, Mann–Whitney *U* test; supplementary table S8, Supplementary Material online). These findings are not consistent with a scenario of hard selective sweeps, in which a new advantageous haplotype is fixed in population quickly, resulting in reduced diversity and Tajima’s *D* within populations and elevated *F_ST_* between populations (Maynard Smith and Haigh 1974). Instead, the observed pattern of genetic variation could be explained by selection acted on multiple haplotypes, for example, polygenic selection (Flood and Hancock 2017). The two methods (*SweepFinder*2 and iHS) identified largely independent gene sets in each group (supplementary table S9, Supplementary Material online). Similar patterns, showing little overlap among candidate genes identified by different methods, have also been observed in other species (Evans et al. 2014; Liu et al. 2017). This may be due to distinct signals of selection captured by different methods, or to low power to detect adaptive evolution, causing little overlap among methods. GO enrichment analyses revealed that candidate genes identified by iHS in groups NOR, UTA, and WES were enriched in defense response, phosphorylation and multiple molecular functions (FDR = 0.042–3.90 × 10^−8^, supplementary table S7, Supplementary Material online). No enrichment was found for candidate genes in the COL population group (FDR > 0.05; supplementary table S7, Supplementary Material online).

## Discussion

### Relaxed Negative and Positive Selection in *B. stricta*

We found evidence for both relaxed negative and positive selection in *B. stricta*. Up to 40% of polymorphic 0-fold sites are effectively neutral (*N_e_s* < 1) in *B. stricta*. The proportion of neutral non-synonymous mutations in *B. stricta* is much higher than in its outcrossing relatives, for example, *Arabidopsis lyrata* (16.5%; Paape et al. 2018), *Arabidopsis arenosa* (∼20%; Monnahan et al. 2019), *C. grandiflora* (13.6%; Williamson et al. 2014), and also higher than that in *A. thaliana* (∼25%; Gossmann et al. 2010), a widely-distributed inbreeding relative. The relaxed purifying selection in *B. stricta* may reflect its long-term small effective population size (*N_e_* = 5.48 × 10^4^, 6.18 × 10^4^, 2.75 × 10^4^, 3.80 × 10^4^ for COL, UTA, NOR, and WES, respectively; see Materials and Methods), pre-dominately self-pollinating breeding system (Song et al. 2006) and the restricted distribution of each population group in western North American (Rushworth et al. 2011; Wang et al. 2019a). Previous work found significant effects of *N_e_* on the DFE; species with large *N_e_* tend to be under pervasive purifying selection (Gossmann et al. 2010). In agreement with this expectation, two *B. stricta* groups (COL and UTA) have low proportions of neutral non-synonymous mutations and relatively large *N_e_*, whereas the northern population groups (NOR and WES) have relatively higher proportions of effectively neutral non-synonymous mutations and also small *N_e_* (fig. 2*A*).

The long-term small *N_e_* also may have contributed to the low estimated rate of adaptive evolution in *B. stricta.* Low rates of adaptive evolution are expected in small populations because of limited input of advantageous mutations, less efficient selection, and strong genetic drift (Gossmann et al. 2010). In contrast, higher rates of adaptive evolution are expected in larger populations because a greater number of advantageous mutations can arise quickly, and selection is more effective (Gossmann et al. 2010). Such relationships between *N_e_* and adaptive evolution have been documented in plant species, and high rates of adaptive evolution were only detected in a few plants with large *N_e_* (Gossmann et al. 2010; Strasburg et al. 2011; Williamson et al. 2014; Lin et al. 2018; Monnahan et al. 2019; Liang et al. 2021). For example, relatives of *B. stricta*, such as *A. arenosa* (*N_e_* = 4.11 × 10^5^, *α* = 0.40; Monnahan et al. 2019) and *C. grandiflora* (*N_e_* = 3.92 × 10^5^, *α* = 0.41; Williamson et al. 2014), showing high rates of adaptive evolution, have *N_e_* an order of magnitude higher than *B. stricta* (*N_e_* = 2.75 × 10^4^−6.18 × 10^4^, *α* = −0.190 to −0.001; assuming *θ* = 4*N_e_μ* and a mutation rate *μ* = 7 × 10^−9^ per site per year for all species).

The estimated rate of adaptation also may be affected by other confounding factors, including gBGC, recombination rate, the presence of slightly deleterious and weakly beneficial alleles, demographic history, population structure, gene duplication, and selection on synonymous variants. We found strong evidence that gBGC has led to relaxed selection in *B. stricta*. Similar to *A. thaliana* and *A. lyrata* (Hämälä and Tiffin 2020), the WS (“GC-biased”) sites have higher proportion of effectively neutral mutations and lower proportion of adaptive substitutions than GC-conservative (“GC-unbiased”) sites. Based on GC-conservative sites, negative selection in all *B. stricta* population groups (34−40% non-synonymous mutations with *N_e_s* < 1) is relaxed in comparison with *A. thaliana* (<20%); the estimated *α* and *ω* values in COL (*α* = 0.12 and *ω* = 0.04) and UTA (*α* = 0.10 and *ω* = 0.04) are comparable with those in *A. thaliana* (*α* = 0.12 and *ω* = 0.02) and *A. lyrata* (*α* = 0.11 and *ω* = 0.02) (Hämälä and Tiffin 2020), but still lower than other outcrossing species (Gossmann et al. 2010; Strasburg et al. 2011; Williamson et al. 2014; Lin et al. 2018; Monnahan et al. 2019; Liang et al. 2021). Therefore, both negative and positive selection are relaxed in *B. stricta*, in comparison with its relatives, after controlling for effects of gBGC.

It is expected that selection is more effective in genomic regions of high-recombination rate where the interference between selected loci is less prevalent due to frequent recombination (Hill and Robertson 1966; Felsenstein 1974). Previous studies in different systems revealed mixed evidence regarding the efficacy of selection and recombination rate. A study in *Drosophila* revealed that both positive and negative selection were stronger in regions with a high recombination rate (Campos et al. 2014), while research in two bird species found that regions with high recombination rates showed higher efficacy of negative selection but not positive selection (Corcoran et al. 2017). In domesticated rice, the negative correlation between recombination rate and polymorphism suggested that efficacy of selection was limited in regions of low recombination rate (Flowers et al. 2011). In contrast, the efficacy of selection was not correlated with recombination rate in *A. thaliana* (Slotte et al. 2011) and wild rice (Flowers et al. 2011). By comparing the strength of selection among genomic regions with different recombination rates, we found evidence for the effect of recombination rate on efficacy of selection in COL but not in other groups. A limitation of our analysis is the coarseness of the genetic map, which may have limited our ability to detect subtle differences of selection among genomic regions. To fully examine the relationship between recombination rates and efficacy of selection in *B. stricta*, higher resolution maps are needed. Nevertheless, the estimated efficacy of negative and positive selection in *B. stricta* are lower than other outcrossing species in all genomic regions regardless of the recombination rate.

Both slightly deleterious and weakly beneficial alleles contribute to polymorphisms, leading to biased estimates of the rate of adaptation (Uricchio et al. 2019). Although the approaches we used to estimate *α* are robust to the presence of slightly deleterious alleles, they are sensitive to polymorphic, weakly beneficial alleles (Eyre-Walker and Keightley 2009; Messer and Petrov 2013; Uricchio et al. 2019). Weakly beneficial alleles could affect our estimates of adaptive rates in two ways. First, weakly beneficial alleles take a long time to fixation, hence contribute substantially to segregating polymorphisms, causing a downward bias in estimates of *α* (Uricchio et al. 2019). Second, when adaptation is weak, background selection may eliminate a substantial portion of weakly beneficial alleles linked to deleterious loci and prevents them from fixation, resulting in substantial underestimation of *α*. Quantitative genetic analyses revealed that a majority of analyzed traits are highly polygenic in *B. stricta* (Anderson et al. 2011, 2014; Lee et al. 2017; Yan et al. 2021), indicating small fitness benefits of adaptive alleles. Widespread background selection in *B. stricta* is supported by the inverse relationship between genetic diversity and gene density (Spearmen’s *ρ* = −0.36 to −0.26, *P* < 0.001 for all windows; *ρ* = −0.35 to −0.24, *P* < 0.001 for windows sampled every 100 kb to account for autocorrelation along the genome; supplementary fig. S3, Supplementary Material online) and reduction of diversity around genes (supplementary fig. S4, Supplementary Material online). Therefore, the low estimates of *α* may reflect a substantial contribution of weakly beneficial alleles to the frequency spectrum, and background selection may have reduced the rate of adaptation in *B. stricta*. In other species, limited evidence for adaptive evolution may be also explained by weakly beneficial alleles and background selection. A recent study in human populations revealed that adaptation was mainly contributed by weakly beneficial variants, and obtained an estimated *α* nearly twice the previous estimates by controlling for effects of background selection and weakly beneficial alleles (Uricchio et al. 2019).

Population demography also can result in biased estimates of *α*, but may not be the major factor in *B. stricta*. Population contraction may cause underestimation of *α*, whereas population expansion can lead to an overestimate of adaptive evolution (Eyre-Walker and Keightley 2009). To account for the effects of demography, we applied two approaches, DFE-alpha and asymptoticMK to estimate *α* by modeling changes of population size and fitting an asymptotic curve to the observed SFS, respectively. We obtained similar results by using these two methods. Additionally, all *B. stricta* groups have experienced recent population expansion (Wang et al. 2019a), which could result in upward bias of estimated adaptation rates. Studies to jointly infer the demographic and selection parameters (Johri et al. 2020) may accurately assess the strength of selection in natural populations. However, this approach is still too simple to incorporate complex models and large datasets, making it difficult to compare with previous genome-wide estimates.

Population structure can decrease the fixation probability of advantageous mutations, and skew the SFS toward rare alleles (Whitlock 2003). *Boechera stricta* occupies heterogeneous habitats and has limited gene flow among geographical areas, causing high population differentiation (Song et al. 2006; Wang et al. 2019a) and frequent local adaptation (Prasad et al. 2012; Lee et al. 2017; Carley et al. 2021; Yan et al. 2021). Locally adapted mutations may not have been fixed within populations, which may partially explain our low estimates of adaptive evolution in *B. stricta*. However, by simulating two subpopulations connected by different levels of gene flow, Gossmann and colleagues (2010) found that *α* may be correctly estimated or overestimated by DFE-alpha. Future study is warranted to assess the effects of population structure on the estimates of selection under more complex models.

Gene duplication is common and local adaptation involving duplicated genes has been reported in *B. stricta* (Prasad et al. 2012). Unfortunately, accurate assessment of variation in duplicated regions is challenging using short read sequencing data, thus we may have missed signatures of adaptive evolution in duplicated genes. Future studies using long-read sequencing and functional genomic analyses are needed to explore the local adaptation in duplicated genes in more detail. In this study, we assumed that mutations of 4-fold sites from non-conserved genomic regions are neutral. If our demographic model were inferred from synonymous SNPs that experienced selection, we would underestimate both purifying selection and positive selection (Gossmann et al. 2010; Johri et al. 2020). There is evidence of non-neutral evolution on synonymous sites because of direct selection on codon usage (Ingvarsson 2008) or linked selection (e.g. Resch et al. 2007; Pouyet et al. 2018). However, strong selection on synonymous codon usage is expected in species with large effective population size (Subramanian 2008), whereas *B. stricta* has long-term small *N_e_* (Wang et al. 2019a). Additionally, the fact that we observed highest polymorphisms at 4-fold sites suggests that synonymous mutations are under weaker selection than their non-synonymous counterparts. Therefore, the relaxed positive and negative selection observed in *B. stricta* is not likely the result of selection on synonymous variants.

### Local Adaptation in *B. stricta*

Despite globally relaxed selection across the genome in *B. stricta*, strong selection was observed in local genomic regions. Interestingly, we found different sets of candidate genes experiencing selective sweeps in each *B. stricta* group. This result suggests a model of local adaptation in *B. stricta*, although we cannot exclude the possibility that species-wide selection may be too weak to be detected in all groups. However, given the same strength of selection across the species range, global sweeps detected in NOR and WES would be identified in COL and UTA, because we have more power to detect selection in COL and UTA groups due to their larger population size. It is expected that populations in contrasting environments, connected by limited gene flow, would adapt to local environments (Hedrick 2006). On the contrary, adaptive introgression could also serve as a driver of local adaptation (Suarez-Gonzalez et al. 2018; Leroy et al. 2020). The low levels of gene flow among populations of *B. stricta* (Song et al. 2006; Wang et al. 2019a), together with heterogeneous environments across the species range (Lee and Mitchell-Olds 2011; Rushworth et al. 2011) would facilitate the evolution of locally adapted genotypes. A significant contribution of ecological factors to genetic differentiation suggests local adaptation in *B. stricta* populations (Lee and Mitchell-Olds 2011), and reciprocal transplant experiments have found evidence for local adaptation at the QTL level in *B. stricta* (Anderson et al. 2013, 2014; Carley et al. 2021). Previous studies also have revealed many adaptive traits associated with local adaptation in *B. stricta*, such as anti-herbivore defense (Prasad et al. 2012; Carley et al. 2021), flowering time (Anderson et al. 2011; Lee et al. 2017; Yan et al. 2021) and leave morphologies (Lee and Mitchell-Olds 2013).

## Conclusion

In summary, we found globally relaxed negative and positive selection in *B. stricta*, which could be explained by its long-term small effective population size. The effects of *N_e_* on the efficacy of selection was also supported by the findings that the WES and NOR groups have smaller *N_e_*, lower efficacy of purifying selection and positive selection compared with the UTA and COL groups. The gBGC and recombination rate have affected the efficacy of selection, but alone cannot explain evidence of relaxed selection in *B. stricta*. Strong selection was detected in local genomic regions and acted on different sets of genes across the species’ range, suggesting local adaptation in *B. stricta*. These results suggested strong effects of *N_e_*, nucleotide composition and recombination rate on the strength of selection, and important roles for local adaptation in driving genetic divergence in *B. stricta*.

## Materials and Methods

### Sampling, Genotyping, and Filtering

We extracted short reads (about 5× average coverage) and genotype data of 467 *B. stricta* accessions from a previous study (Wang et al. 2019a, 2019b). These accessions represent four previously-identified genetic groups (Wang et al. 2019a), comprising 157, 126, 105, and 79 accessions from groups COL, UTA, NOR, and WES, respectively (supplementary fig. S1, Supplementary Material online). Information regarding sequencing and genotyping is detailed in Wang et al. (2019a) and briefly described below. Raw reads were trimmed using Trimmomatic v0.39 (Bolger et al. 2014) and mapped to the *B. stricta* reference genome (Lee et al. 2017) using BWA v0.7.17 (Li 2013). The reference genome is an assembly of the LTM genotype (WES group). This assembly comprises 1990 scaffolds with an *N*50 size of 2.19 Mb and a total length of 189.35 Mb (Lee et al. 2017). Two hundred and eight scaffolds (totally 183.72 Mb, 97% of the assembled genome) were placed on seven linkage groups; 27,416 genes were annotated, and the completeness of annotation was 96.40% (BUSCO score) (Lee et al. 2017). All individuals showed high mapping rates (96.21–98.65%; supplementary table S1, Supplementary Material online). In addition, the WES group showed lower genetic diversity than COL and UTA in most site categories (*W* ranges from 342,686 to 54,531,298, *P* < 2e^−16^, Mann–Whitney *U* tests with Bonferroni correction; fig. 1 and supplementary table S2, Supplementary Material online). These results suggested limited reference bias when using LTM as a reference genome to call SNPs in *B. stricta*.

SNPs were called using HaplotypeCaller implemented in GATK v4.1 (McKenna et al. 2010), and filtered by stringent criteria to remove low quality data: 1) assigned homozygous genotypes supported by <2 reads as missing; 2) assigned heterozygous genotypes supported by <20 reads or ratio of major/minor depth (number of reads supporting major allele/number of reads supporting minor allele) <0.25 as missing; 3) removed SNPs genotyped in <50% individuals; 4) retained only biallelic SNPs with mean depth <20 and proportion of heterozygous genotypes <15%; 5) discarded indels; and 6) discarded all sites located in annotated repeated regions of the reference genome. Comparisons with Sanger sequences showed that the accuracy of called genotypes approaches 99.88% (Wang et al. 2019a). In addition, validation and sensitivity analyses revealed that genotypes could be called with high accuracy (99.03–99.93%), independent of sequencing depth (2.5×–40×) in *B. stricta* (Wang et al. 2019a). The high accuracy of called genotypes based on relative low sequencing depth in *B. stricta* is most likely due to its extremely low heterozygous rate (*π* = 0.00107–0.00192) as a pre-dominantly inbreeding species (Song et al. 2006; Wang et al. 2019a). Similarly, the average positive rate of called genotypes exceeds 98% based on a sequencing depth of 5× in *A. thaliana* (1001 Genomes Consortium 2016). In this study, we further removed SNPs with missing rate >50% or mean depth >20 or proportion of heterozygous genotypes ≥15% across the 467 selected *B. stricta* accessions, and retained 3,995,289 SNPs for subsequent analyses. The mean depth and missing rate of these SNPs were about 4.5× and 20%, respectively (supplementary fig. S5, Supplementary Material online).

### Identifying Conserved Genomic Regions

GERP++ (Davydov et al. 2010) was used to identify CSs. Briefly, genomes of ten Brassicaceae species were downloaded from previous publications (supplementary table S3, Supplementary Material online), and each genome was soft masked using RepeatMasker v4.0.7 (http://www.repeatmasker.org). Then, a 10-way multiple alignment was obtained following a lastZ/Multiz pipeline previously described (Haudry et al. 2013; Hupalo and Kern 2013) and using *B. stricta* as the reference genome. After that, 4-fold sites in the *B. stricta* genome were extracted from the multiple alignments to reconstruct a phylogenetic tree with branch lengths representing the neutral evolutionary rate among species within the alignment. Finally, GERP++ was used to estimate site-specific constraint score and to discover CSs with a length >12 bp. Candidate CSs were assigned a location category based on annotation of the *B. stricta* genome. In cases where a long CS extends across different categories, it was split into different sub-segments according to genome annotation, and only sub-segments with length >12 nt were retained.

### Estimating Summary Statistic

We applied a probabilistic method in ANGSD v0.930 (Korneliussen et al. 2014) to calculate summary statistics, including genetic diversity (*π*), Tajima’s *D* (Tajima 1989), Fay & Wu’s *H* (Fay and Wu 2000) in each population group of *B. stricta*, and relative genetic differentiation (Weir and Cockerham’s weighted *F_ST_*) (Weir and Cockerham 1984) and absolute divergence (*d_XY_*) (Nei 1987) for each pair of groups. To account for mutation rate variation across the genome, we calculated the relative node depth (Feder et al. 2005) by dividing *d_XY_* of each group pair with their mean divergence to an outgroup species, *Boechera retrofracta*. The Fay & Wu's *H* was estimated based on unfolded SFS polarized by two outgroup species, *B. retrofracta* and *Capsella rubella*. All other summary statistics were estimated on folded SFS. We only considered high-quality sites passing the filtering criteria above (see Sampling, genotyping, and Filtering), as well as filtering thresholds in ANGSD: 1) reads with mapping quality >30 (-minMapQ); 2) bases with quality score >30 (-minQ); 3) SNPs with *P* value < 1 × 10^−4^ (-snp_pval). The inbreeding coefficient of each individual was obtained from Wang et al. (2019a) and incorporated into the calculation of SFS. Per-site diversity was calculated based on genotype likelihoods, and then averaged for each 20-kb window. For intra-population summary statistics, we only considered sites with at least 50% of individuals genotyped in that population. For inter-population summary statistics, we used sites with at least 50% of individuals successfully genotyped in both populations. We also calculated *π* and *F_ST_* based on called genotypes, which are highly correlated with those estimated from genotype likelihoods (Pearson’s correlation coefficient *r* = 0.956−0.974, *P* < 2.2e^−16^ for *π*; *r* = 0.983−0.996, *P* < 2.2e^−16^ for *F_ST_*; supplementary figs S6 and S7, Supplementary Material online). Therefore, we only report results estimated based on genotype likelihoods using ANGSD. The long-term effective population size (*N_e_*) was estimated as *θ*/4*μ*, where *θ* is synonymous polymorphism and *μ* is the mutation rate per generation (Tajima 1989). Assuming a generation time of 2 years and mutation rate of 7 × 10^−9^ substitutions per year (Ossowski et al. 2010), we calculated the synonymous polymorphisms on 4-fold sites, and converted them to *N_e_* for the four *B. stricta* groups (*N_e_* = 5.48 × 10^4^, 6.18×10^4^, 2.75×10^4^, 3.80×10^4^ for COL, UTA, NOR, and WES, respectively).

### Distribution of Fitness Effects and Proportion of Adaptive Substitutions

We classified sites into different categories based on annotations of the *B. stricta* genome (Lee et al. 2017), and calculated SFS and divergence for each category of sites. DFE-alpha v2.16 (Keightley and Eyre-Walker 2007; Eyre-Walker and Keightley 2009) was implemented to estimate the DFE of new mutations, the proportion of adaptive substitutions (*α*), and the relative rate of adaptive substitution (*ω*) for each category of sites. Because the presence of slightly deleterious mutations can downwardly bias estimates of positive selection (Eyre-Walker and Keightley 2009), we predicted the numbers of neutral and slightly deleterious substitutions based on estimated DFE, and then compared them with the observed number of substitutions. The 4-fold degenerate sites outside conserved genomic regions were used as the neutral reference, with *B. retrofracta* as outgroup to calculate between-species divergence. We examined three demographic models: 1) one-epoch with constant effective population size (*N_e_*); 2) two-epoch with a single change in *N_e_*; 3) three-epoch with two historical *N_e_* changes. The Akaike Information Criterion indicated that two-epoch was the best-fitting model in all groups (supplementary table S10, Supplementary Material online). Since methods implemented in DFE-alpha assume a random mating population and recommend using the same number of alleles for all sites, we performed a downsampling procedure (Keightley and Eyre-Walker 2007). To maximize the number of segregating sites, we randomly sampled 78, 63, 52, and 39 individuals from COL, UTA, NOR, and WES groups, respectively, for each site. Sites with sample size less than the threshold in any group were discarded. Because *B. stricta* is predominantly inbreeding, we phased genotypes using Beagle v4.1 (Browning and Browning 2007) with default settings, and then randomly chose one haplotype per individual. We also excluded SNPs from the ∼10% of genomic regions (Chromosome 1: 15.3–24.4 Mb, Chromosome 5: 0.47–3.4 Mb, and Chromosome 7: 0.45–5.1 Mb) under balancing selection (Wang et al. 2019a). To generate 95% confidence intervals of estimated parameters for each site category, we divided the genome into 20-kb windows and estimated parameters based on 200 bootstrap replicates. Significance of differences between site categories and genetic groups was determined through Mann–Whitney *U* tests, and *P*-values were corrected using the Bonferoni adjustment method (Whitlock and Scluter 2008).

We further applied an asymptotic MK analysis to estimate *α* (Messer and Petrov 2013) using the web-based asymptoticMK tool (Haller and Messer 2017). This approach estimated *α* for different frequency classes separately and then calculated an asymptotic estimate of *α* (*α*_asym_) by fitting to the empirical data (Messer and Petrov 2013; Haller and Messer 2017). The asymptoticMK is robust to the presence of deleterious mutations and recent demographic events (Messer and Petrov 2013; Haller and Messer 2017). We calculated substitution rates and derived allele frequencies for test and control regions based on the same data used for DFE-alpha. The polymorphism levels were grouped into the same number of frequency bins for test and control regions.

To investigate whether the efficacy of selection was affected by gBGC, we divided synonymous (4-fold degenerate) and non-synonymous (0-fold degenerate) sites into three categories: SW, WS, and GC-conservative (SS + WW). In each category, the first letter represents the ancestral allele and the second letter represents the derived allele. The letter “S” represents alleles G or C with strong hydrogen bonds, while the letter “W” represents allele A or T with weak hydrogen bonds. To define the ancestral state, we used genotype information of two outgroup species *B. retrofracta* and *C. rubella*. Ancestral state was defined as the allele shared by *B. stricta* and at least one outgroup.

To investigate how recombination rate affected the strength of selection across the genome, we divided GC-conservative sites into three bins of different recombination rate, and estimated DFE, *α* and *ω* using DFE-alpha for each bin. We considered only GC-conservative sites to control the effects of gBGC on the efficacy of selection. To estimate the recombination rate (cM/Mb) across the genome, we used the genetic map from Lee et al. (2017). This genetic map was created based on a cross between LTM (the reference genome, WES group) and SAD12 (COL group), with a resolution of 20-kb. We mapped GC-conservative sites to the genetic map. About 99.5% of GC-conservative sites were successfully aligned to the genetic map. The recombination rate of each 20-kb window was assigned as the recombination rate for all GC-conservative sites within that window. Because there is an inverted region (1.7 −10.3 Mb on Chromosome 1) without resolution on the genetic map, we excluded GC-conservative sites in this region from data analysis. We divided GC-conservative sites into three equal-size bins with low (<3.2 cM/Mb), middle (3.2 to 6.4 cM/Mb), and high (>6.4 cM/Mb) recombination rate (supplementary table S5, Supplementary Material online).

We also considered new methods to jointly infer demographic and selection parameters (Johri et al. 2020). Here, we have chosen not to employ this approach because it uses a small fraction of the genome, it has only been tested with simple single-population models, and it is difficult to compare with previous studies. Because *B. stricta* has low *N_e_* (Wang et al. 2019a), it is plausible that our populations have high proportions of mutations that are effectively neutral. Furthermore, our estimates from GERP++ do not suffer from interactions between estimates of demography and selection addressed by Johri and colleagues.

### Detecting Signatures of Selection

To investigate signals of selection in *B. stricta* we first scanned the genome using *SweepFinder2* (DeGiorgio et al. 2016) with grid sizes 1, 5, 10, 20, and 50 kb. Results from different grid sizes were consistent, thus we only show results with grid size of 20 kb. To avoid biases when inferring the ancestral allelic states, we used folded SFS for each group. We calculated the empirical frequency spectrum based on all SNPs (-f), and then estimated CLR for each grid across the genome. Grids located in genomic regions with low coverage (<5 kb per 20 kb non-overlapping sliding window) were excluded from data analyses. We also identified regions under positive selection across the genome using a haplotype-based test, integrated haplotype score (iHS) (Voight et al. 2006) as implemented in *selscan* v1.2.0a (Szpiech and Hernandez 2014). The iHS value was calculated for each SNP with MAF >0.05 and then normalized in frequency bins across the entire genome. SNPs located in genomic regions with low quality (coverage < 5 kb per 20 kb window) were discarded.

Because population demography may introduce false positives to *SweepFinder2* and iHS analyses, we modeled the demographic history of *B. stricta* to determine the significance of estimates. First, we used *fastsimcoal2* (Excoffier et al. 2013) to simulate 10,000 1-Mb fragments under the previously estimated best demographic model for *B. stricta* (Wang et al. 2019a). The best model is a four-population isolation-with-migration model, where each group experienced two steps of population size changes after splitting (Wang et al. 2019a). Parameters of the demographic model and settings for simulation are provided in a supplementary data S1, Supplementary Material online. We estimated the CLR and iHS on simulated data using the same parameters as for the real data, and corrected *P*-values for multiple testing using Benjamini–Hochberg FDR adjustment (Benjamini and Hochberg 1995). We used a cutoff of FDR < 0.01 to determine the significant estimates. We finally considered 318–471 grids (FDR < 0.01) corresponding to 3.5−5.1% of all tested grids as candidate swept regions in *SweepFinder2*, and 1,078–2,533 outlier SNPs (0.18−0.50% of all tested SNPs; FDR < 0.01) as candidates under selection in iHS analyses.

For both *SweepFinder2* and iHS analyses, genes within 10-kb flanking regions of candidate grids (or SNPs) were regarded as candidate genes under selection. The web-based agriGO v2.0 tool (Tian et al. 2017) was used for gene ontology (GO) enrichment analyses based on annotation of *B. stricta* genome (Lee et al. 2017). The significance (*P*-values) of over-representation of functional classes of genes was calculated using Fisher’s test and then corrected with Benjamini–Hochberg FDR (Benjamini and Hochberg 1995) for multiple tests. GO terms with FDR < 0.05 were considered as significantly enriched.

## Supplementary Material

evac043_Supplementary_Data

## Data Availability

Previously published whole-genome sequencing data are available at the GenBank under accession numbers: SRP054739, SRP134283-SRP134373, SRP134393-SRP134433, SRP134436-SRP134479, SRP134481-SRP134572, and SRP134581-SRP134671. All SNPs used in population genetic analyses and locations of all accessions are available in the Dryad Data Archive at https://doi.org/10.5061/dryad.574pc6n.
