# Supplementary material for: Globally Relaxed Selection and Local Adaptation in Boechera stricta
Source: Genome Biol Evol. 2022 Mar 29;14(4):evac043. doi: 10.1093/gbe/evac043 (PMC9011030; doi:10.1093/gbe/evac043)
Supplement: evac043_Supplementary_Data [file evac043_supplementary_data.docx]

**Supporting information**

**Table S1** The 467 whole genome re-sequenced *Boechera stricta* accessions analyzed in this study.

**Table S2** Genetic diversity (π ± standard error, in unit of 10^-3^) on different categories of sites in each group.

**Table S3** Ten Brassica species used for identifying conserved sequences in *B. stricta*.

**Table S4** Estimates (95% confidence interval) of distribution of fitness effects (DFE) of new amino acid mutations, proportion of fixation driven by positive selection (α) and the relative rate of adaptive substitution (ω) in different site categories of four *B. stricta* groups. (a) Comparisons were made among categories of sites in each group. (b) Comparisons were made among four groups for each category of sites.

**Table S5** Estimates (95% confidence interval) of distribution of fitness effects (DFE) of new amino acid mutations, proportion of fixation driven by positive selection (*α*) and the relative rate of adaptive substitution (ω) in genomic regions of different recombination rate. Comparisons were made among genomic regions in each group.

**Table S6** Comparison of summary statistics (mean ± standard error) between the sweep regions (identified by *SweepFinder*2) and the rest of the genome, for all groups. *P*-values were estimated by Mann–Whitney *U* test.

**Table S7** GO categories overrepresented for candidate genes under selection (identified by *SweepFinder*2 and iHS).

**Table S8** Comparison of summary statistics (mean ± standard error) between the sweep regions (identified by iHS) and the rest of the genome for all groups. *P*-values were estimated by Mann–Whitney *U* test.

**Table S9** The number of candidate genes overlapping between *SweepFinder2* and iHS.

**Table S10** Relative likelihood of the three models investigated in DFE-alpha. Each model was examined on different categories of sites in each group.

**Fig. S1 Genetic variation of *Boechera stricta*.** (A) Geographic distribution of the 467 accessions representing the four genetic groups of *B. stricta.* (B) Genetic structure of samples detected by Admixture (*K* = 4). (C) Relative genetic differentiation (*F*_ST_; lower triangle) and absolute divergence (*d*_XY_; upper triangle) between each pair of groups, and genetic diversity (π) within each group (diagonal line) estimated in 20-kb sliding windows across the genome. (A) and (B) are adapted from Fig. 1 in Wang *et al.* (2019).

**Fig. S2 Fraction of conserved sites in the *B. stricta* genome.** (A) Percentage of conserved elements in coding (CDS), Intergenic, Intronic, 5’ UTR (Utr5) and 3’ UTR (Utr3) regions. (B) Proportion of conserved sites for different sets of regions of the *B. stricta* genome. Region categories comprise coding regions (CDS), 5’ UTR, 3’ UTR, 30-bp intronic regions flanking exons (IntronM), middle of introns (IntronC) and intergenic regions (bases are assigned according to physical distance to the closest gene).

**Fig. S3** **Correlation between nucleotide diversity and gene density per 20kb window.** In each plot, correlation and significance are tested with two-sided Spearman’s rank correlation test.

**Fig. S4** **Relative nucleotide diversity versus distance to the nearest gene in *B. stricta* population groups.** Lines depict a loess curve (span of 0.3) and shading represents bootstrap-based 95% confidence intervals.

**Fig. S5 Comparisons of mean depth (A) and missing rate (B) across different categories of sites in *Boechera stricta*.** In these box plots, the median is shown by a horizontal line, while the bottom and top of each box represents the first and third quartiles. The whiskers extend to 1.5 times the interquartile range. Outliers are not shown in the plot. IntronC, middle region after excluding the first and last 30 bp of each intron; IntronM, the first and last 30 bp of each intron.

**Fig. S6 Correlation between genetic diversity (π) estimated from genotype likelihoods and from called genotypes.** Correlation and significance are tested with Pearson’s correlation test (two sided) in each group. Each dot represents a 20-kb window.

**Fig. S7 Correlation between population differentiation (***F*_ST_**) estimated based on genotype likelihoods and on called genotypes.** Correlation and significance are tested with Pearson’s correlation test (two sided) in each group. Each dot represents a 20-kb window.

**Data S1** Parameters of the demographic model simulated in *fastsimcoal2*.
